# Beamforming Transmission in IEEE 802.11ac under Time-Varying Channels

**DOI:** 10.1155/2014/920937

**Published:** 2014-07-24

**Authors:** Heejung Yu, Taejoon Kim

**Affiliations:** ^1^Department of Information and Communication Engineering, Yeungnam University, Gyeongsan 712-749, Republic of Korea; ^2^School of Information and Communication Engineering, Chungbuk National University, Cheongju 361-763, Republic of Korea

## Abstract

The IEEE 802.11ac wireless local area network (WLAN) standard has adopted beamforming (BF) schemes to improve spectral efficiency and throughput with multiple antennas. To design the transmit beam, a channel sounding process to feedback channel state information (CSI) is required. Due to sounding overhead, throughput increases with the amount of transmit data under static channels. Under practical channel conditions with mobility, however, the mismatch between the transmit beam and the channel at transmission time causes performance loss when transmission duration after channel sounding is too long. When the fading rate, payload size, and operating signal-to-noise ratio are given, the optimal transmission duration (i.e., packet length) can be determined to maximize throughput. The relationship between packet length and throughput is also investigated for single-user and multiuser BF modes.

## 1. Introduction

As wireless data traffic increases explosively, cellular networks cannot meet the increasing demands, and data offloading with a wireless local area network (WLAN) has been considered the best solution. Along with this trend, a new amendment to the WLAN standard, called IEEE 802.11ac, has been under development [1]. Thus, IEEE 802.11a [2], IEEE 802.11n [3], and IEEE 802.11ac are called the legacy, high throughput (HT), and very high throughput (VHT) WLAN, respectively. In VHT-WLAN, 256-QAM modulation, the extension of bandwidth up to 160 MHz, and multiple-input multiple-output (MIMO) transmission up to 8 streams are defined to provide 6.9 Gbps data rate. Additionally, the standard includes closed-loop beamforming (BF) schemes including single-user BF (SU-BF) and multiuser BF (MU-BF) methods to improve spectral efficiency with a given channel condition. For these BF transmissions, an access point (AP) sends a sounding packet including only preambles and receives a compressed beam frame with modified downlink channel information from stations (STAs). In particular, in MU-BF mode, an AP can transmit multiple data streams simultaneously to multiple STAs without causing interference by using the transmit beams. This MU-BF is a new feature only in IEEE 802.11ac. However, the closed-loop approaches require sounding overhead, although they achieve higher spectral efficiency. Hence, the throughput gain of BF methods over the open-loop schemes will be obtained only when packet size is longer than a certain value to compensate for the loss due to sounding overhead. When channels are static, throughput increases as the payload size in a packet becomes longer, because the relative portion of sounding overhead to data payload decreases. In [4–6], the comparison of open-loop transmission and BF schemes, including SU-BF and MU-BF, was investigated under static channels.

In practice, wireless channels vary over time due to the mobility of a transmitter, a receiver, and reflectors. The time variation of channels is one of the main causes of deterioration in performance of closed-loop BF schemes. The delay between sounding time and transmission time causes the mismatch between beam and channel. The mismatch then causes the loss of spectral efficiency. This loss of spectral efficiency increases as the length of a packet increases. Hence, the sounding overhead and the channel mismatch result in a tradeoff in terms of throughput, and there exists the optimal length of a packet with a given fading rate.

In this paper, we analytically investigate throughput, considering the sounding procedure and channel variation. Based on the analytical result, we obtain the optimal packet size and verify it with numerical results. Depending on signal-to-noise ratio (SNR), the speed of mobile STAs, payload size, and BF schemes (including SU-BF and MU-BF), we examine the behaviors of the effective throughput of IEEE 802.11ac systems.

We make use of the standard notational conventions. Vectors and matrices are written in boldface, with matrices in capitals. All vectors are column vectors. For a matrix **A**, **A** ^*T*^ and **A** ^*H*^ indicate the transpose and Hermitian transpose of **A**, respectively. For scalar *a*, a* is the complex conjugate of *a*. 0 and **I** are all-zero and identity matrices with an appropriate size. For a vector **a**,  ||**a**||  denotes the norm. The notation **x** ~ *N*(***μ***, Σ) means that vector **x** is a Gaussian random vector with a mean vector ***μ*** and a covariance matrix Σ.

This paper is organized as follows.  Section 2 includes the system model for a WLAN with an AP and multiple STAs. The frame structures and protocols of IEEE 802.11ac related to BF modes are discussed in Section 3. In Section 4, we investigate the effects of time-varying channels in SU-BF and MU-BF modes.  Section 5 includes the analytical throughput results and some observations about them. In Section 6, we verify the analysis with numerical results. Finally, Section 7 concludes the paper.

## 2. System Model

**Figure 1:**
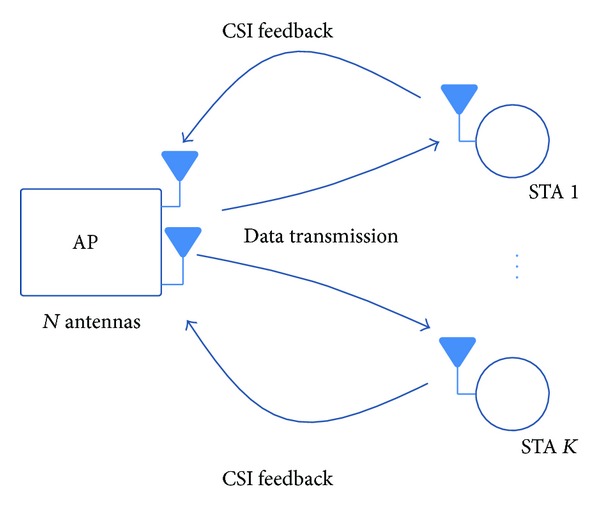
System model for an MU-BF with one AP and *K* STAs.

We consider the WLAN system model including one AP with *N* antennas and *K* (≤*N*) STAs equipped with a single antenna as shown in Figure 1. In this paper, we assume a single antenna at STAs because most STAs are equipped with one antenna due to space and cost limitations in practice. However, we can simply extend our results to cases where STAs have multiple antennas. In SU-BF mode, the AP sends the packet to one STA after channel sounding. On the other hand, the AP transmits a data packet with multiple destinations after receiving the channel information from the STAs. The transmit signal for SU-BF mode at time *n* is given by

(1) $$\begin{array}{c} x\left(n\right)=vs\left(n\right), \end{array}$$

where *s*(*n*) is a transmit data symbol with unit power. **v** (**v** ^*H*^ **v** = 1) denotes a BF vector that does not change over time. The design criterion for SU-BF is to maximize the achievable rate. To this end, the beam vector is determined by

(2) $$\begin{array}{c} v=\frac{H^{H}}{\sqrt{HH^{H}}}, \end{array}$$

where **H** is a channel matrix with a size of 1 × *N* from an AP to a STA. It is called a maximal ratio transmission (MRT) beam design. If a channel matrix is time-varying, the optimal beam vector is also changed to guarantee optimality. However, the exact channel information is required to track the optimal beam vector, but it is not possible. Therefore, the beam vector obtained with the channel during the sounding step is used until the next channel sounding time, and then we cannot avoid the rate loss.

With MU-BF, we have another objective for the beam design. Since data symbols with multiple destinations are transmitted simultaneously, interference with other STAs should be precancelled by using the beam vectors. The transmit signal at an AP for MU-BF is expressed by

(3) $$\begin{array}{c} x\left(n\right)=\overset{K}{\underset{k=1}{\sum }}v_{k}s_{k}\left(n\right), \end{array}$$

where **v**_*k*_ and *s*_*k*_(*n*) denote a transmit beam vector and a data symbol for STA *k*. This design goal can be written as

(4) $$\begin{array}{c} H_{j}v_{k}=0,\;\forall j\neq k. \end{array}$$

With a definition of the cumulative interfering channel matrix ${\tilde{H}}_{k}={\left[H_{1}^{T}\cdots H_{k-1}^{T}H_{k+1}^{T}\cdots H_{K}^{T}\right]}^{T}$, the above condition can be rewritten as

(5) $$\begin{array}{c} {\tilde{H}}_{k}v_{k}=0. \end{array}$$

Hence, the beam vector for STA *k* is in the null space of ${\tilde{H}}_{k}$. This is known as a zero-forcing (ZF) approach, which shows the best performance under a noise-free condition by cancelling the interference leakage to other users. As an alternative for noisy channel conditions, a regularized ZF method that enhances the desired signal by allowing interference less than a certain threshold was proposed in [7]. Sadek et al. introduced a beam design method based on the signal-to-leakage-plus-noise ratio (SLNR) [8]. Its extension to the time-varying channel was proposed by Yu and Lee [9]. In this paper, we employ the ZF approach to simplify the analysis. Even with MU-BF, the performance loss due to time variation in channels cannot be avoided. Because the beam vectors determined during the sounding step are not in the null space of the cumulative interfering channel matrices, the interference leakage to STAs is inevitable and will cause the rate loss. One of the significant issues in MU-BF transmission is scheduling of users. The effective SNR of each STA significantly depends on the cumulative channel matrix given by $\overset{-}{H}={\left[H_{1}^{T}\cdots H_{K}^{T}\right]}^{T}$. When the cumulative channel matrix is well conditioned, that is, the condition number of $W={\overset{-}{H}\overset{-}{H}}^{H}$ is small, the effective SNRs for all STAs are large and then high throughput can be achieved. On the other hand, if a cumulative channel has a large condition number and is ill-conditioned, an effective SNR of one STA is small, and we cannot avoid throughput loss. Here, a condition number for **W** is defined as a ratio between the largest and smallest eigenvalues of **W**. If a condition number is small, channel vectors from an AP to STAs are nearly orthogonal. Then, the orthogonal project of a channel vector for a STA onto the null space of the cumulative interfering channel matrix has large norm, and the effective SNR also becomes large. If a condition number is large, however, the directions of all channel vectors are similar, and then the orthogonal project has a small norm. Therefore, the performance of MU-BF transmission highly depends on the scheduling of STAs, that is, the selection of STAs to transmit data simultaneously. By choosing STAs with a smaller condition number for the cumulative channel matrix, we can expect more throughput gain by using MU-BF mode over SU-BF.

To analytically examine the effects of channel variation, we employ the Gauss-Markov channel model given by

(6) $$\begin{array}{c} h\left(n\right)=\beta h\left(n-1\right)+u\left(n\right),\;n=1,2,\ldots , \end{array}$$

where *β* stands for the fading coefficient. Also, *h*(0) ~ *N*(0,1) and *u*(*n*) ~ *N*(0,1 − *β* ^2^) [10–12]. In Rayleigh fading channels, the fading coefficient is determined as follows:

(7) $$\begin{array}{c} \beta =J_{0}\left(2\pi f_{d}T_{s}\right)=\overset{\infty }{\underset{r=0}{\sum }}\frac{{\left(-1\right)}^{r}}{2^{2r}{\left(r!\right)}^{2}}{\left(2\pi f_{d}T_{s}\right)}^{2r}, \end{array}$$

where *f*_*d*_ and *T*_*s*_ are the maximum Doppler frequency and the unit of time index, respectively. *J*_0_(·) is the zeroth order Bessel function of the first kind. In channel matrices, all elements are independently and identically modelled by (6).

## 3. Sounding Procedure and Frame Structure

**Figure 2:**
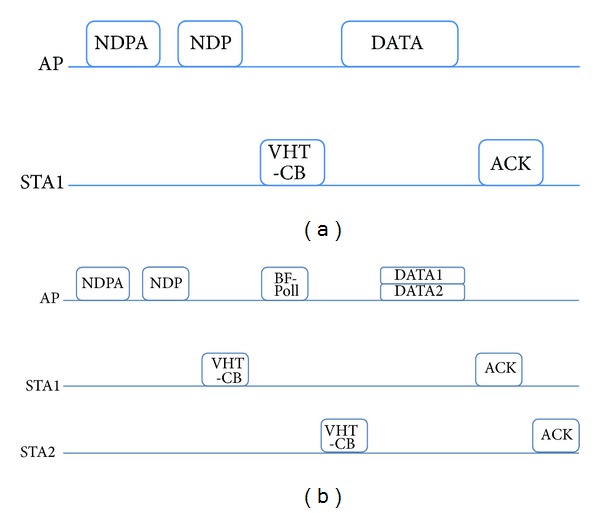
Frame-exchange sequences for (a) SU-BF and (b) MU-BF.

**Figure 3:**
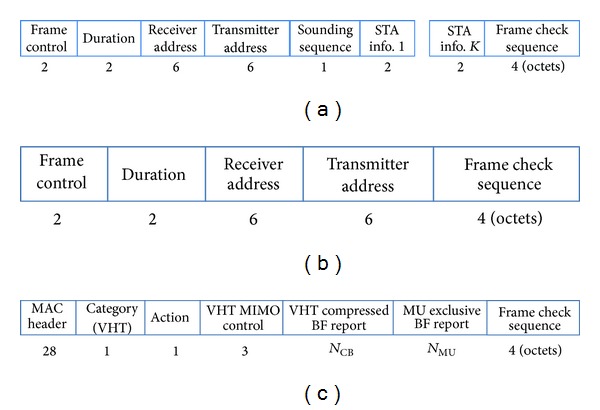
Frame contents for (a) NDPA, (b) BF-Poll, and (c) VHT-CB frames. (The number below each box denotes the number of bytes for each field.)

To evaluate the throughput of BF methods in IEEE 802.11ac, we summarize the sounding process and frame structure. For the initial transmission to gain an opportunity to access a channel, carrier sensing during a distributed coordinated function interframe space (DIFS) time and a backoff process are required. Here, the backoff counter is randomly chosen in an interval [0, CW − 1], where CW is the size of a contention window. Therefore, the average of an initial channel access overhead can be assumed by

(8) $$\begin{array}{c} T_{\text{dcf}}=T_{\text{DIFS}}+\frac{{\text{CW}}_{\min}-1}{2}T_{\text{slot}}, \end{array}$$

where *T*_DIFS_ and *T*_slot_ are the DIFS time and slot time in IEEE 802.11ac. Additionally, CW_min⁡_ denotes the minimum contention window, and this value is the initial value used for channel access. When packet collision is detected, the value of a contention window increases exponentially. It is called the exponential backoff mechanism. Therefore, (8) is the average channel access overhead with an assumption of no collision. When the total number of associated STAs is small and a small control packet like a null data packet announcement (NDPA) frame for initial channel access is considered, the probability of packet collision is small enough to overlook the collision. For SU-BF and MU-BF modes, feedback of CSI from the STAs is required. For this purpose, IEEE 802.11ac defines additional control and management frames including a null data packet (NDP), NDPA, beamforming report poll (BF-Poll), and VHT-compressed beamforming (VHT-CB) frames. On downlink, the beamformer that stands for an AP sends an NDPA frame to initiate the sounding process of obtaining the information of beamformees, denoting the target STAs. With an interval of short interframe space (SIFS) time, the AP sends an NDP frame without any data as a pilot frame for the STAs to estimate the downlink channels. The target STAs receive the NDP frame, estimate the channel matrix, and transform the channel matrix into compressed form with a sequence of angles. This compressed channel information is fed back to the AP with a VHT-CB frame. For SU-BF mode with a single STA, the AP sends the beamformed data frame by using the reconstructed CSI from the compressed channel information. On the other hand, MU-BF requires additional information from multiple beamformees. The sequence of feedback is determined by the order of STA information in the NDPA frame. After the AP receives the first VHT-CB frame, it sends a BF-Poll frame indicating the next STA to transmit a VHT-CB frame. After gathering all VHT-CB frames, the AP transmits a beamformed data frame with multiple data streams to the associated STAs. If the STAs correctly decode the data, they send back the acknowledgement (ACK) frame to the AP in the same order of VHT-CB transmission. All frame-exchange sequences for SU-BF and MU-BF modes are illustrated in Figure 2. In addition, the contents of control information in NDPA, BF-Poll, and VHT-CB frames are shown in Figure 3.

Based on such operation scenarios, we can calculate the throughput of each beamforming method. For simplicity, we assume that all control frames, including NDPA, BF-Poll, and ACK frames, use the 6 Mbps transmission rate with binary phase shift keying (BPSK) modulation and a 1/2 code rate in the legacy format. The transmission rate of the VHT-CB frame is determined with the highest rate at which the channel can support the legacy format. In IEEE 802.11ac, the channel matrix is compressed with a sequence of angles of Givens rotation matrices, which diagonalize a given channel matrix. As an example, we assume a 1 × 3 channel matrix (3-antenna AP, 1-antenna STA). The channel matrix **H** = [*h*_1_ *h*_2_ *h*_3_] is normalized and Hermitian transposed into **v** given by (2). Then, **v** = [*v*_1_ *v*_2_ *v*_3_]^*T*^ is decomposed into four angles of Givens rotation as follows:

(9) $$\begin{array}{c} \phi_{11}=∠\left(v_{1}\right)-∠\left(v_{3}\right),\\ \phi_{21}=∠\left(v_{2}\right)-∠\left(v_{3}\right),\\ \psi_{21}=\text{ta}{\text{n}}^{-1}\left(\frac{\left|v_{2}\right|}{\left|v_{1}\right|}\right),\\ \psi_{31}=\tan^{-1}\left(\sqrt{\frac{{\left|v_{3}\right|}^{2}}{{\left|v_{1}\right|}^{2}+{\left|v_{2}\right|}^{2}}}\right). \end{array}$$

Here, {*ϕ*_*ij*_} are the relative phase differences between components and {Ψ_*ij*_} represent the relative amplitudes. *ϕ*_*ij*_ ∈ [0, 2*π*] is quantized with 4, 6, 7, or 9 bits, whereas Ψ_*ij*_ ∈ [0, *π*/2] is quantized with 2, 4, 5, or 7 bits. Additionally, the average SNR of total used tones is required for SU-BF, and the deviation of per-tone SNR relative to average SNR is also required for MU-BF. The SNR information with a range from −10 dB to 53.75 dB is quantized with 8 bits. In Figure 3(c), the VHT-compressed BF report field includes the sequence of Givens angles and average SNR information, and the MU-exclusive-BF report field contains the per-tone SNR deviations for the subset of subcarriers. Therefore, the total length of the VHT-CB frame depends on the quantization of angle and SNR as well as the number of used tones. We can reduce the length of VHT-CB frame by using a grouping method. In this case, however, the channel information becomes inaccurate. Therefore, the accuracy of channel information is a cost to reducing the length of VHT-CB frame. In this paper, we assume that the maximum number of quantization bits (i.e., 9 bits for {*ϕ*_*ij*_} and 7 bits for {Ψ_*ij*_}) is used, and the grouping method is not used to minimize the inaccuracy of the CSI. Therefore, the VHT-compressed BF report field includes 8*N*_*C*_ + (7 + 9)*N*_*S*_ *N*_*a*_/2 bits, where *N*_*C*_, *N*_*S*_, and *N*_*a*_ denote the number of transmit streams, used tones, and angles for compression, respectively. The first term is the number of bits for the average SNR, and the second is for the quantized angles. Moreover, the MU-exclusive-BF report includes 4*N*_*S*_ *N*_*C*_ bits for SNR deviation for the subset of subcarriers. Hence, under the system model considered in this paper, that is, *N*_*C*_ = 1, *N*_*S*_ = 52 (20 MHz channel bandwidth), *N*_*a*_ = 2 (2-antenna AP, 1-antenna STA), and *N*_*S*_ = 30, the total number of bytes for the VHT-compressed BF report and MU-exclusive-BF report fields is *N*_CB_ = 105 and *N*_MU_ = 15 bytes, respectively.

**Figure 4:**
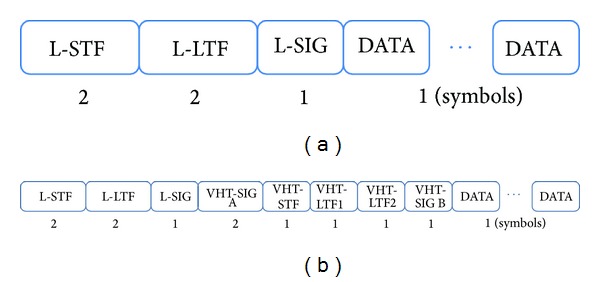
(a) Legacy frame format and (b) VHT frame format. (The number below each box denotes the number of OFDM symbols.)

IEEE 802.11ac has two different frame formats, including legacy and VHT frames, as shown in Figure 4. Because IEEE 802.11ac devices should coexist with legacy IEEE 802.11a devices and IEEE 802.11n HT devices, control frames such as NPDA, BF-Poll, and ACK frames should be in legacy format. The duration of a legacy frame is determined by

(10) $$\begin{array}{c} T_{\text{leg}}=T_{\text{L-PRE}}+T_{\text{L-SIG}}+T_{\text{ofdm}}\left\lceil \frac{22+8L}{N_{\text{DBPS}}}\right\rceil , \end{array}$$

where the parameters are defined as follows:

- *T*_L-PRE_ is the transmit time for the legacy short and long training fields (16 *μ*s);
- *T*_L-SIG_ is the transmit time for the legacy SIGNAL field (4 *μ*s);
- *T*_ofdm_ is one orthogonal frequency division multiplexing (OFDM) symbol duration (4 *μ*s);
- *N*_DBPS_ is the number of data bits per OFDM symbol;
- L is the number of bytes of MAC contents, including MAC overhead and payload.

Here, 22 bits in the last term in (10) represent the SERVICE and TAIL bits. The durations of control frames are given by

(11) TNDPA=TL-PRE+TL-SIG+Tofdm⌈22+8(22+2K)24⌉={56 μs,SU-BF60 μs,MU-BF  with  K=2TBF-Poll=TL-PRE+TL-SIG+Tofdm⌈22+16024⌉=52 μs,TACK=TL-PRE+TL-SIG+Tofdm⌈22+11224⌉=44 μs,

where *N*_DBPS_ is assumed to be 24 for the lowest data rate mode.

The transmission rate of VHT-CB frames is determined with the highest rate the channel can support in VHT format. The frame format for VHT-CB frames is not defined explicitly [1]. To reduce the time for VHT-CB transmission (i.e., sounding overhead), we assume that VHT-CB frames are transmitted in VHT format. To evaluate the length of a VHT-CB frame, we examine the duration of a VHT frame. Consider

(12) TVHT=TL-PRE+TL-SIG+TVHT-PRE+TVHT-SIG+Tofdm⌈22+8LNDBPS⌉,

where

- *T*_VHT-PRE_ is transmit time for VHT short and long training fields;
- *T*_VHT-SIG_ is transmit time for VHT SIGNAL A and B field (12 *μ*s).

Then, the duration of a VHT-CB frame with *N*_CB_ = 105 bytes and *N*_MU_ = 15 bytes is given by

(13) TVHT-CB=TL-PRE+TVHT-SIG+TVHT-PRE+TL-SIG+Tofdm⌈22+1256NDBPS⌉.

The modulation and coding scheme (MCS) level of a VHT-CB frame is determined by the maximum rate that can be supported by a given channel condition. Additionally, the length of a data packet for SU-BF mode is determined by

(14) TDATASU-BF=TL-PRE+TL-SIG+TVHT-PRE+TVHT-SIG+Tofdm⌈22+8(LMO+Ldata)NDBPS⌉,

where *L*_MO_ and *L*_data_ are the numbers of bytes for MAC overhead and data payload, respectively. For MU-BF, on the other hand, the length of a data packet is determined by

(15) TDATAMU-BF=TL-PRE+TL-SIG+TVHT-PRE+TVHT-SIG+Tofdm(max⁡i=1,2⁡⌈22+8(LMO+Ldata,i)NDBPS,i⌉),

where *L*_data,*i*_ and *N*_DBPS,*i*_ denote the number of bytes for data and the number of bits per OFDM symbol for the *i*th STA. In MU-BF, the multiple data streams are simultaneously transmitted, and the length of a data frame depends on the maximum number of OFDM symbols of all STAs.

## 4. Performance Analysis in Time-Varying Channels

In time-varying channels, an achievable rate for a beamforming scheme deteriorates because of channel delay and channel fading. Additionally, imperfect channel information due to limited feedback can cause some loss in an achievable rate. However, this rate loss is ignored in this paper, because these effects do not change over time and can be reduced by increasing the resolution of channel quantization.

For SU-BF, we can derive the rate loss due to mobility. To obtain the effective SNR under a static channel, we rewrite the received signal at time *m* as follows:

(16) y(m)=Hx(m)+n(m)=HHHHHHs(m)+n(m).

Hence, the effective SNR is given by

(17) $$\begin{array}{c} \gamma_{\text{SU-BF}}^{\text{static}}=\frac{HH^{H}}{\sigma^{2}}=\frac{{||H||}^{2}}{\sigma^{2}}. \end{array}$$

Under time-varying channels, the beam vector is determined with the feedback information from a STA. Therefore, we have to consider the feedback delay, which is a time difference between the channel estimation time at a STA and the packet transmission time at an AP. Assuming that the channel is estimated at time 0 and the feedback delay is *r*, the received signal at a STA is given by

(18) y(r)=H(r)HH(0)H(0)HH(0)s(r)+n(r)=(a)(βrH(0)+W(r))HH(0)H(0)HH(0)s(r)+n(r)=βrH(0)HH(0)s(r)+W(r)HH(0)H(0)HH(0)s(r)+n(r),

where the equality (*a*) holds by the channel model and **W**(*r*) (=[*w*_1_(*r*),…, *w*_*N*_(*r*)]) is obtained from (6). The *i*th element of **H**(*r*) is given by

(19) hi(r)=βhi(r−1)+ui(r)=β[βhi(r−2)+ui(r−1)]+ui(r) ⋮=βrhi(0)+∑p=1r−1βpui(r−p)=βrhi(0)+wi(r).

Hence, the distribution of *w*_*i*_(*r*) is the complex Gaussian with a zero mean and a variance of *σ*_*v*_^2^(*r*) = 1 − *β* ^2*r*^. Then, the effective SNR is given by

(20) $$\begin{array}{c} \gamma_{\text{SU-BF}}^{\text{varying}}\left(r\right)=\frac{\beta^{2r}{||H\left(0\right)||}^{2}}{\sigma_{v}^{2}\left(r\right)+\sigma^{2}}. \end{array}$$

The rate loss is expressed as follows [13]:

(21) LSU-BF(r)=log⁡⁡(1+γSU-BFstatic)−log⁡⁡(1+γSU-BFvarying(r))≤log⁡⁡(γSU-BFstaticγSU-BFvarying(r))=log⁡⁡(1−β2r+σ2β2rσ2),

where the inequality holds since log⁡⁡(·) is a concave and increasing function. As the SNR increases, the value of the rate loss approaches this upper bound.

On the other hand, the received signal and effective SNR at STA *i* for MU-BF in the static channel are written as

(22) yi(m)=Hi(∑k=1Kvksk(m))+n(m)=Hivisi(m)+∑k=1,k≠iKHivksk(m)+n(m)=Hivisi(m)+n(m),

(23) $$\begin{array}{c} \gamma_{\text{MU-BF}}^{\text{static}}=\frac{{\left|H_{i}v_{i}\right|}^{2}}{\sigma^{2}}, \end{array}$$

respectively.

When a channel is time-varying and feedback delay is *r*, the received signal and its effective SNR are changed into

(24) yi(r)=Hi(r)vi(0)si(r) +∑k=1,k≠iKHi(r)vk(0)sk(r)+n(r)={βrHi(0)+Wi(r)}vi(0)si(r) +∑k=1,k≠iK{βrHi(0)+Wi(r)}vk(0)sk(r)+n(r)=βrHi(0)vi(0)si(r)+Wi(r)vi(0)si(r) +∑k=1,k≠iKWi(r)vk(0)sk(r)+n(r),

(25) $$\begin{array}{c} \gamma_{\text{MU-BF}}^{\text{varying}}\left(r\right)=\frac{\beta^{2r}{\left|H_{i}\left(0\right)v_{i}\left(0\right)\right|}^{2}}{K\sigma_{v}^{2}\left(r\right)+\sigma^{2}}, \end{array}$$

respectively. Hence, the upper bound of the rate loss for the total number of STAs in MU-BF mode is

(26) $$\begin{array}{c} L_{\text{MU-BF}}\left(r\right)\leq K\log\left(\frac{K\left(1-\beta^{2r}\right)+\sigma^{2}}{\beta^{2r}\sigma^{2}}\right). \end{array}$$

## 5. Throughput Analysis

In terms of sounding overhead, we can achieve higher throughput with the longer data packet under the static channels. However, the longer packet causes the channel information mismatch due to the feedback delay. Hence, throughput loss is inevitable. Therefore, the optimal packet length according to the tradeoff between sounding overhead and rate loss due to mobility must be obtained with their analytical formulas. With SNR given by *σ* ^2^ and initial channel realization **H**(0), we can find the throughput depending on the number of data bits in different transmission schemes.

At first, we evaluate the throughput for SU-BF mode with one DATA and ACK frame exchange according to the sounding process given as

(27) RSU=8LdataTdcf+TNDPA+TNDP+TVHT-CB+4TSIFS+TACK+TDATASU-BF.

Additionally, the sum throughput for two STAs associated with the MU-BF transmission is calculated as

(28) RMU=8(Ldata,1+Ldata,2)(Tdcf+TNDPA+TNDP+TBF-Poll+2TVHT-CB+7TSIFS+2TACK+TDATAMU-BF).

In both equations, the values of  *T*_VHT-CB_, *T*_DATA_^SU-BF^, and *T*_DATA_^MU-BF^ are determined by the effective SNR of each transmission mode. The MCS index of the corresponding packet determines the value of *N*_DBPS_. Additionally, the possible values for the spectral efficiency and the required SNR to decode the packet with the given MCS index in IEEE 802.11ac are shown in Table 1. The required SNR is obtained by the Shannon capacity formula given by log⁡⁡(1 + SNR), and there is a gap from the practical SNR value under the various conditions, including loss of actual coding and modulation schemes from the channel capacity formula, channel power profile, packet length, and implementation loss including channel estimation and synchronization error. To show the analytical performance, however, we use the values in the table. In static channels, the longer packet can achieve the higher throughput. At a given bit error rate, the longer packet has the higher packet error rate. However, channel coding schemes like turbo code and low density parity check code (LDPC) show the lower bit error rate when the size of a packet is large. Therefore, we can assume that these two positive and negative effects compensate for each other, and packet error rate does not depend on the size of packets. However, the main focus in this paper is the throughput under time-varying channels, where the longer packet is encoded with a lower MCS index, that is, a lower rate due to the rate loss caused by channel mismatch.

To evaluate throughput performance under time-varying channels, we need the delay of channel information for each mode. The time difference between the end of an NDP packet reception and the end of the data packet reception can be considered channel feedback delay. In SU-BF mode and MU-BF mode supporting 2 STAs, the feedback delay values are given as

(29) $$\begin{array}{c} r_{\text{SU}}=2T_{\text{SIFS}}+T_{\text{VHT-CB}}+T_{\text{DATA}}, \end{array}$$

(30) $$\begin{array}{c} r_{\text{MU}}=4T_{\text{SIFS}}+2T_{\text{VHT-CB}}+T_{\text{BF-Poll}}+T_{\text{DATA}},\;\; \end{array}$$

respectively. With these delay values and mobile STAs' speed, we can estimate the rate loss of each mode according to (21) and (26). Since these losses increase with the channel feedback delay, there exists a tradeoff between sounding overhead and rate loss due to delay, and the optimal length of a packet can be obtained. In detail, we can calculate the spectral efficiency with the initial channel **H**(0) or **H**_*i*_(0) and obtain the reduced spectral efficiency as a function of delay. Since the transmitter will use the highest MCS index for which the required SNR is less than the reduced spectral efficiency, we can determine the values of *N*_DBPS_. As we can see in the next section, throughput is a concave function of payload size, and then there exists the optimal payload size, which maximizes throughput under a given condition. Therefore, we can achieve maximum throughput by fragmenting the actual payload with multiple packets containing payloads of the optimal size under a given condition. If the optimal payload size is larger than the traffic amount in a queue, we have to transmit data with one data packet.

On the other hand, the value of *N*_DBPS_ for VHT-CB frames transmitted with an omnidirectional mode is determined with the following SNR value:

(31) $$\begin{array}{c} \gamma_{\text{VHT-CB}}=\frac{{||H||}^{2}}{2\sigma^{2}}. \end{array}$$

Here, we assume that the rate loss due to channel variation can be ignored because the VHT-CB frames are not beamformed* but transmitted in an open-loop manner*.

The problem formulation to find the optimal frame length for the SU-BF mode is given by

(32) Ldataopt=argmax⁡Ldata RSU(NDBPS(γMCS))  s.t. γ(Ldata)≥γMCS,

where *γ*(*L*_data_) and *γ*_MSC_ mean the SNR value with feedback delay to transmit *L*_data_ bytes of payload and the required SNR to support a given MCS level, respectively. Then, *N*_DBPS_(*γ*_MCS_) denotes the number of data bits per OFDM symbol corresponding to the MCS level satisfying the constraint. Since this problem includes the discrete values representing the MSC level, the closed form solution does not exist. The throughput is a concave function of the size of payload *L*_data_. Hence, *L*_data_^opt^ is easily found if the optimal MCS level, that is, *N*_DBPS_(*γ*_MCS_), is obtained with a given *L*_data_. Therefore, we suggest an algorithm to find the optimal MCS to transmit  *L*_data_  payload as shown in Algorithm 1.

*N*_DBPS_(*γ*_MSC_) = 0 means that SNR is too low to transmit a packet, even with the lowest MSC. We only show an SU-BF case, but the extension to MU-BF can be developed simply.

## 6. Numerical Results

**Figure 5:**
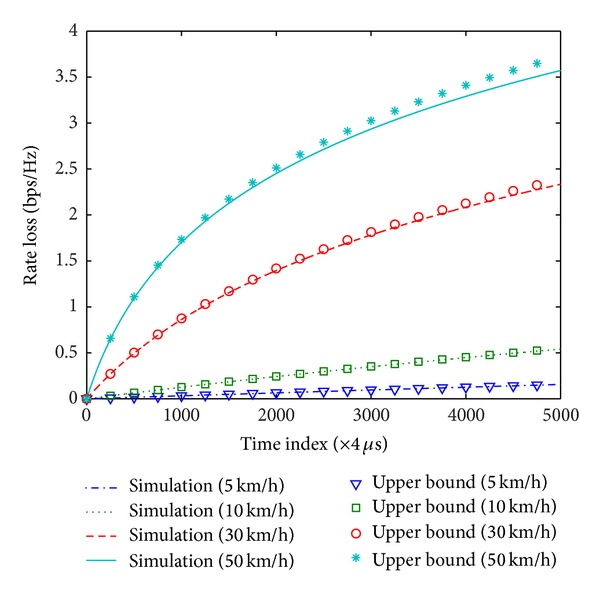
Rate loss due to channel variation in SU-BF mode.

**Figure 6:**
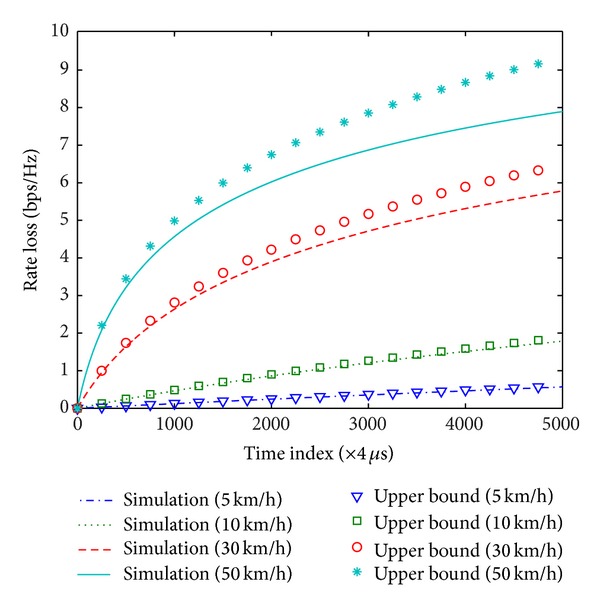
Sum rate loss of two STAs due to channel variation in MU-BF mode.

In this section, the analysis of rate loss in time-varying channels and throughput of beamforming schemes is verified. For computer simulations, we generate 2 × 2 complex matrices for which elements follow a complex Gaussian distribution with zero mean and unit variance. The carrier frequency is 5.8 GHz and the time unit is 4 *μ*s, which is the duration of an OFDM symbol in IEEE 802.11ac. All results are obtained with a 20 MHz channel bandwidth. To realize time-varying channels, we use the Gauss-Markov model with fading coefficient given by (7). Figures 5 and 6 show the rate loss due to the variation of channels in SU-BF and MU-BF modes, respectively. Here, SNR is assumed to be 20 dB. As shown in the figures, the upper bound can be used as a reasonable approximation of the rate loss when the channel varies slowly and the time index (i.e., the feedback delay) is small.

**Figure 7:**
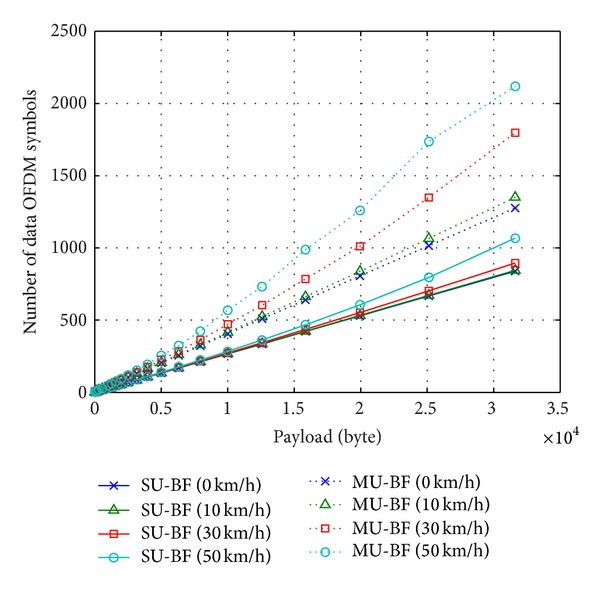
Optimal packet length with a given payload and mobile speeds (solid lines: SU-BF and dotted lines: MU-BF, SNR = 20 dB).

Next, we determine the optimal payload length with the highest MCS index that can be supported by a given time-varying channel condition. If the selected MCS level is too high to be supported, the packet cannot be decoded by the receiver, and it is retransmitted until the packet is successfully received. On the other hand, if the MCS level is too low, the duration of a packet transmission is unnecessarily long, and throughput is reduced. Therefore, determination of the optimal MCS and packet length is one of the significant factors in achieving the highest throughput. The variation in the number of data OFDM symbols as a function of the number of bytes in a data payload depending on mobile speed is shown in Figure 7. Here, we also assume that SNR is 20 dB. The optimal length is obtained with the algorithm in Section 5 by averaging results of 1000 i.i.d. channel realizations. As mobile speed increases, the optimal number of data OFDM symbols with a given data payload becomes larger since the rate loss increases with mobile speed. In MU-BF mode, the optimal number of data OFDM symbols is determined by the channel condition of the worst STA. Therefore, the increasing ratio of the optimal number of data OFDM symbols in MU-BF is faster than SU-BF.

In some cases where the effective SNR of the worst user is too low to support the lowest MCS, MU-BF transmission is not possible. Such cases are ignored in Figure 7.

**Figure 8:**
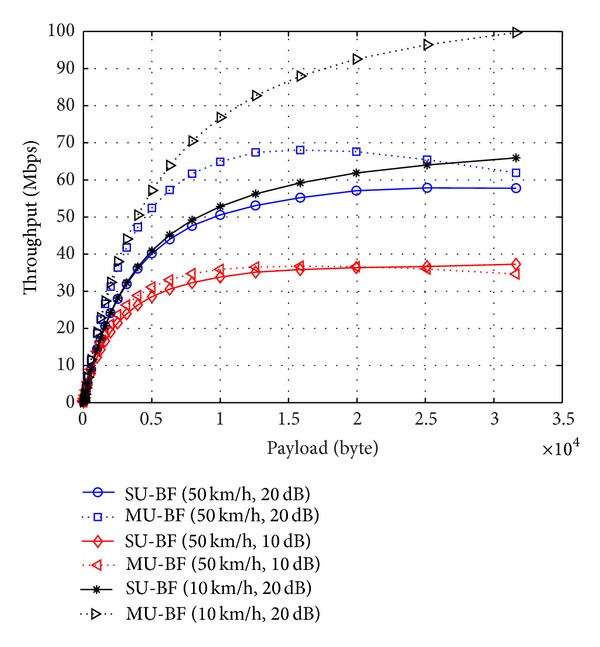
Throughput of SU-BF and MU-BF modes with a given SNR and mobile speeds (solid lines: SU-BF and dotted lines: MU-BF).

We also compare the throughput of SU-BF and MU-BF modes with different mobile speeds. In simulations, we assume that an AP transmits the same number of payloads to 2 STAs. In the SU-BF case, the AP transmits a beamformed packet to each STA with the frame-exchange sequence, as shown in Figure 2(a). For MU-BF, the AP sends the packet to 2 STAs simultaneously, as shown in Figure 2(b). In general, SU-BF mode is preferred when the amount of data to be sent is small and the channel varies rapidly. When the amount of transmit data is large and channel varies slowly, MU-BF can achieve higher throughput. These observations can be verified with the numerical results. Moreover, we can see that throughput is a concave function of payload size. In cases where SNR is high and channels vary slowly, throughput increases monotonically with payload size. In other cases, throughput is a concave function, and there exists an optimal payload size that maximizes throughput, as shown in Figure 8.

**Figure 9:**
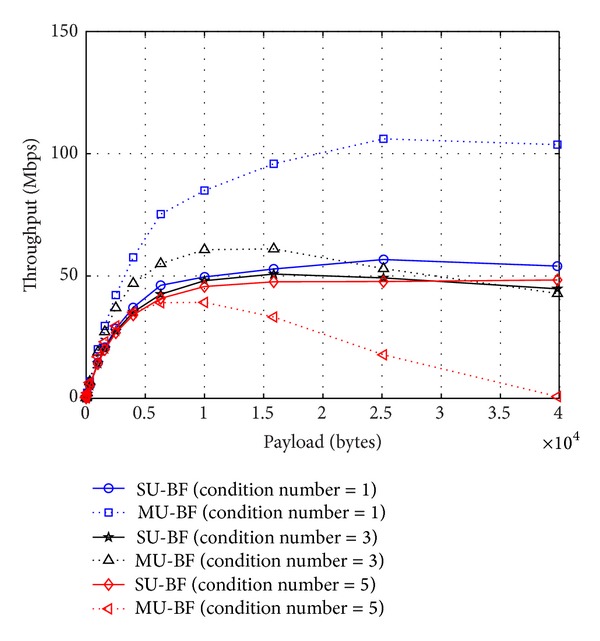
Throughput of SU-BF and MU-BF modes under channels with a given condition number of $W={\overset{-}{H}\overset{-}{H}}^{H}$ (SNR = 20 dB and mobile speed = 50 km/h).

Finally, the condition number of an initial channel matrix also has a strong impact on throughput behaviors of SU-BF and MU-BF modes. On well-conditioned channels, that is, channels with a small condition number, MU-BF transmission is more efficient since both effective SNR values of the 2 STAs are high, and the duration of an MU-BF data frame becomes shorter. On ill-conditioned channels with a high condition number, however, the length of an MU-BF packet is very long, because one STA has a very low effective SNR to satisfy the condition given by (5). This can be examined with the numerical results in Figure 9. When the condition number of $W(={\overset{-}{H}\overset{-}{H}}^{H})$ is 5, MU-BF gains nothing over SU-BF. The importance of user scheduling can be clarified by this result.

## 7. Conclusions

We analyzed throughput under time-varying channels for SU-BF and MU-BF modes under the IEEE 802.11ac WLAN standard. To this end, we derived the rate loss of MRT and ZF beam design approaches for SU-BF and MU-BF modes, respectively. By investigating the special feedback format, sounding protocol, and packet structures, the throughput was analyzed with several assumptions that simplify the analysis. Depending on the payload size, operating SNR, and the speed of mobile STAs, the throughput behaviors of beamforming transmissions were examined with numerical results. Additionally, the importance of user scheduling was also shown, with throughput results from channel conditions having different condition numbers. The main goal of this paper is to give some insight into a mode selection rule in the design of IEEE 802.11ac WLAN systems.

## Figures and Tables

**Algorithm 1:**
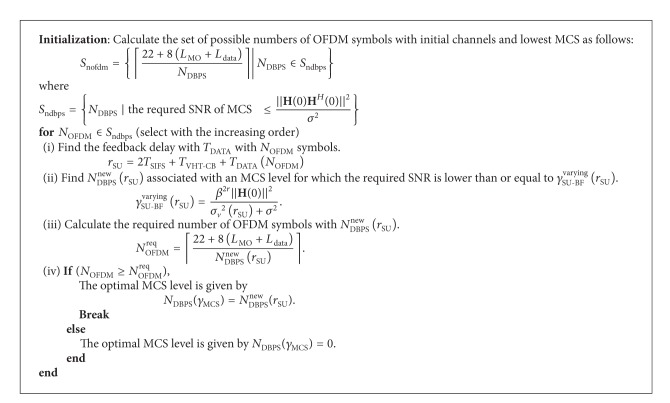
An algorithm for determining the optimal MCS with a given payload size.

**Table 1:** MCS's and required SNR values in IEEE 802.11ac.

MCS index	Modulation	Code rate	*N*_DBPS_	Spectral efficiency (bps/Hz)	Required SNR (dB)
0	BPSK	1/2	26	0.5	−3.83
1	QPSK	1/2	52	1	0
2	QPSK	3/4	78	1.5	2.62
3	16-QAM	1/2	104	2	4.77
4	16-QAM	3/4	156	3	8.45
5	64-QAM	2/3	208	4	11.67
6	64-QAM	3/4	234	4.5	13.35
7	64-QAM	5/6	260	5	14.91
8	256-QAM	3/4	312	6	17.99
9	256-QAM	5/6	Not valid		
